# Construction of a transposase accessible chromatin landscape reveals chromatin state of repeat elements and potential causal variant for complex traits in pigs

**DOI:** 10.1186/s40104-022-00767-3

**Published:** 2022-10-11

**Authors:** Tao Jiang, Ziqi Ling, Zhimin Zhou, Xiaoyun Chen, Liqing Chen, Sha Liu, Yingchun Sun, Jiawen Yang, Bin Yang, Jianzhen Huang, Lusheng Huang

**Affiliations:** State Key Laboratory of Pig Genetic Improvement and Production Technology, Jiangxi Agricultural University, Nanchang, 330045 China

**Keywords:** Chromatin accessibility, Pig, Tissue specific, Transcriptome, Transposable elements

## Abstract

**Background:**

A comprehensive landscape of chromatin states for multiple mammalian tissues is essential for elucidating the molecular mechanism underlying regulatory variants on complex traits. However, the genome-wide chromatin accessibility has been only reported in limited tissue types in pigs.

**Results:**

Here we report a genome-wide landscape of chromatin accessibility of 20 tissues in two female pigs at ages of 6 months using ATAC-seq, and identified 557,273 merged peaks, which greatly expanded the pig regulatory element repository. We revealed tissue-specific regulatory elements which were associated with tissue-relevant biological functions. We identified both positive and negative significant correlations between the regulatory elements and gene transcripts, which showed distinct distributions in terms of their strength and distances from corresponding genes. We investigated the presence of transposable elements (TEs) in open chromatin regions across all tissues, these included identifications of porcine endogenous retroviruses (PERVs) exhibiting high accessibility in liver and homology of porcine specific virus sequences to universally accessible transposable elements. Furthermore, we prioritized a potential causal variant for polyunsaturated fatty acid in the muscle.

**Conclusions:**

Our data provides a novel multi-tissues accessible chromatin landscape that serve as an important resource for interpreting regulatory sequences in tissue-specific and conserved biological functions, as well as regulatory variants of loci associated with complex traits in pigs.

**Supplementary Information:**

The online version contains supplementary material available at 10.1186/s40104-022-00767-3.

## Background

Eukaryotic genomes are usually packed into nucleosomes, which comprise of 147 bp DNA wrapping around a histone octamer, forming the structural units of chromatin. Chromatin is categorized as active euchromatin or inactive heterochromatin based on its accessibility [1, 2], which is linked to fundamental cellular processes including gene transcription, DNA replication and repair [3]. The accessible chromatin often reflects binding of transcriptional machinery to cis-regulatory elements such as promoters and enhancers [4–8]. Profiling the accessible chromatin in different tissues could help to interpret regulatory basis underlying spatiotemporal transcription of genes [9–11], as well as genomic variants causing diseases susceptibility and complex trait variations [12–15].

Over the past decades, several chromatin accessibility profiling methods including DNase I hypersensitive site sequencing (DNase-seq), micrococcal nuclease sequencing (MNase-seq) and assay for transposase-accessible chromatin using sequencing (ATAC-seq) have been developed. Among these, ATAC-seq has become increasingly popular because of its simplicity, high sensitivity and low cell number requirement [16], and has been applied to construct regulatory elements landscapes in human and mouse [17–20]. The international consortiums like, Encyclopedia of DNA Elements (ENCODE) [21], Roadmap Epigenomics projects [22] and the Functional Annotation of Animal Genomes (FAANG) [23] have played important roles in generating the epigenetic data in multiple species. There have been efforts to profile accessible chromatin in pigs [24–26]. However, only few tissues including fat, cerebellum, cortex, heart, liver, muscle, spleen, lung and hypothalamus were covered in these studies. Moreover, studies have investigated chromatin states of TEs including LINE, SINE, LTR and DNA transposons in human and mouse [27–29], allowing better understanding the regulatory function of TEs. However, the chromatin landscape of pig genomic TEs has not been explored yet. In particular, the PERV, as a type of TE, whose sequences are inserted into the pig genome, can be transmitted to humans after xenotransplantation, causing disease risk [30, 31].

Here, we performed ATAC-seq on 40 samples from 20 tissues in 2 female Duroc-Landrace-Yorkshire (DLY) commercial pigs, including 10 tissues (medulla spinalis, bronchia, parotid gland, pharynx, stomach, small intestine (SI), kidney, ovary, cervix, thymus) that have not been investigated in previous studies. We inferred the open chromatin regions displaying tissue-specific or tissue-conserved accessibility. By integrating transcriptomic data (RNA-seq), we revealed open chromatin regions that significantly correlated with gene transcription. In addition, we examined TEs including the PERVs and different TEs in the open chromatin regions, providing insights into the regulatory role of the TEs and their evolutionary history. The data and analyses in this study provide vital resources to explore the function of cis-regulatory elements in determining tissue-specific biological functions and complex traits in pigs.

## Methods and materials

### Sample collection

The pigs used in this study were obtained from a slaughter house in Nanchang, Jiangxi province. 20 tissues (brain, cerebellum, hypothalamus, medulla spinalis, lung, bronchia, parotid gland, pharynx, stomach, small intestine, liver, kidney, cervix, ovary, heart, thymus,* longissimus* *dorsi*, semimembranosus, backfat, leaf fat) was collected from 2 healthy commercial sows at age of 6 months. After slaughter, tissue samples were collected in sterile tube and stored at − 80 °C.

### ATAC-seq library preparation and sequencing

ATAC-seq libraries were prepared as previously described with minor modifications [16]. Briefly, samples were ground to powder with liquid nitrogen using mortar. Then, we homogenized the tissue powder in 2 mL ice-cold homogenization buffer (10% NP40, 500 mmol/L EDTA, 1 mol/L sucrose, 100 mmol/L PMSF, 14.3 mol/L β-mercaptoethanol, 1 mol/L CaCl_2_, 1 mol/L Mg(Ac)_2_, 1 mol/L Tris [pH 7.8], nuclease-free H_2_O) using a 7-mL Dounce homogenizer for 20 strokes. We filtered the solution through a 70-μm cell strainer and gradient centrifugation with different concentrations of iodixanol. After that, approximately 60,000 nuclei from each sample were collected, centrifuged at 500 × *g* for 5 min at 4 °C, washed the pellet with 1 mL ATAC-RSB (5 mol/L NaCl, 1 mol/L MgCl_2_, 1 mol/L Tris-HCl [pH 7.4], nuclease-free H_2_O) containing 0.1% Tween-20, centrifuged at 500 × *g* for 10 min at 4 °C; and resuspended the pellet in 50-μL of tagmentation reaction mix (2 × Tagmentation buffer, 10 × PBS, 0.5% Digitonin, 10% Tween20, Tn5 transposase and nuclease-free H_2_O), incubated for 30 min at 37 °C. Libraries were prepared using active motif kit (ATAC-Seq Kit #53150) as previously described [32]. The ATAC-seq DNA was sequenced in the 150 bp paired-end sequencing mode with a NovaSeq 6000 platform. Two biological replicates were performed per tissue.

### ATAC-seq processing

**Fig. 1:**
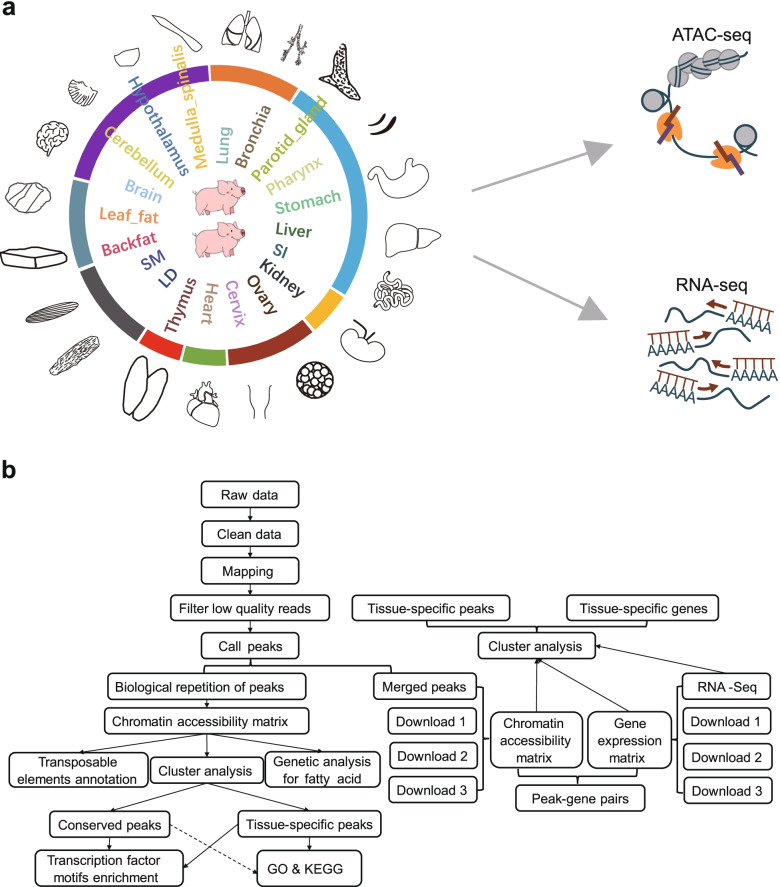
Experimental design and statistical analysis for constructing the landscape of chromatin accessibility. **a** Schematic diagram showing tissues assayed in this study. The ring colors represent different organ systems. Starting with the location of the brain tissue, clockwise around the circle, corresponding to the nervous system (brain, cerebellum, hypothalamus, medulla spinalis), respiratory system (lung, bronchia), digestive system (parotid gland, pharynx, stomach, liver, small intestine (SI)), urinary system (kidney), reproductive system (ovary, cervix), circulatory system (heart), lymphatic system (thymus), locomotor system (*longissimus* muscle (LD), semimembranosus (SM)), endocrine system (backfat and leaf fat). **b** The workflow of analysis in this study. Please refer to the Methods and materials for details

According to the Fig. 1b, the raw data with sequence adapters and low-quality reads were trimmed with Trim Galore v0.6.6 [33] using default parameters. The high-quality reads were then aligned to the pig genome (*Sus scrofa* 11.1) using Bowtie2 v2.3.5.1 [34]. Duplicate alignments were removed using Picard toolkit v1.119 [35] and alignments with low mapping quality (mapq < 30) and mitochondrial DNA were removed with SAMtools v1.9 [36]. We used MACS2 v2.1.1 [37] to generate the accessible chromatin regions (peaks) for each sample with the parameter --qvalue 0.01 --nomodel --shift − 100 --extsize 200 -B -SPMR --keep-dup all, and then we merged peaks across all samples within the same tissue in two individuals overlapping peaks using BEDTools merge [38]. We recalculated the number of reads in each merged peak using SAMtools bedcov [36] and peaks per kilobase million values were determined to measure the peak intensity using R program v3.5.1 [39]. The clustering of the samples based on peak intensity were performed using hcluster function with ward. D method in R program v3.5.1 [39], and visualized using R package circlize [40] and dendextend [41]. Genomic annotation for peaks was performed with Genomic Association Tester [42]. And tissue-specific peaks are defined as peak intensity in specific tissue at least twice higher than in the others.

### RNA-seq library preparation, sequencing and processing

Total RNA was extracted from 40 frozen samples using Trizol and sequenced using MGI-2000 system with paired end and strand specific. RNA-seq reads of each sample were mapped to pig genome (*Sus scrofa* 11.1) using STAR v2.7.1a [43]. Expression level of each gene was determined with StringTie [44] and featureCounts [45] and transcript per million (TPM) normalization was performed using R program. Only genes expressed (TPM > 0) across the 40 samples in more than 2 samples were included in the subsequent analyses. The clustering of the samples based on gene expression levels were performed using hcluster function with ward.D method in R program, and visualized using R package circlize [40] and dendextend [41]. And two samples (stomach_sample1 and backfat_sample2) with poor clustering were discarded. And tissue-specific genes are defined as expression level in specific tissue at least twice higher than in the others.

### Gene ontology enrichment analysis

The gene ontology enrichment analysis was performed using ClueGO [46]. The *P*-values for the enrichment of Gene Ontology (GO) terms were corrected using Benjamini-Hochberg approach.

### Transcription factor motifs

We tested for enrichment of vertebrates known transcription factor motifs in peaks using HOMER [47] with default parameters.

### Correlation of peaks with gene expression

To clarify the regulatory relationship between peaks and genes, three other data were used in this study. The first study was from Kern et al. [26]. A total of 16 samples were collected from 8 tissues (fat, cerebellum, brain, hypothalamus, liver, lung, muscle and spleen) of yorkshire with 2 biological replicates per tissue. The second was designed by Zhao et al. [48], which include 5 tissues (muscle, liver, fat, spleen, heart) of MeiShan, LargeWhite, Enshi and Duroc with 1–2 biological replicates per tissue. And the last one was executed by Yang et al. [49], which we downloaded and only included one tissue (skeletal muscle) of Luchuan and Duroc pigs with 3 biological replicates per breed (Additional file 1: Table S3). In total, combined with our own data, there were 85 samples of ATAC-seq and corresponding RNA-seq. After the above uniform ATAC-seq process analysis, we identified 796,506 peaks with *P*-value less than 10^−5^, and 14 samples with poor tissue clustering were discarded. And then 527,718 peaks exist in at least 2 samples were retained. For RNA-seq, after uniform RNA-seq process analysis above, 24,974 genes were identified, then, we selected 17,634 genes with TPM > 1 in at least 10% samples for further analysis. Using 527,718 peaks and 17,634 genes across 71 samples, we predicted target genes for peaks by implementing correlation between peak intensity and gene expression. Genes whose transcription start sites (TSSs) were within 500 Kb from the center of peaks were considered. We identified 827,942 significant correlations at *q*-value less than 0.01.

### Spatial correlation between peaks and TEs

The data of TEs from pig (*Sus scrofa* 11.1) were analyzed by RepeatMasker v2.9.0 [50] with parameter -s. And correlated interval sets between peaks and TEs were calculated by GenometriCorr (Genometric Correlation) [51] which was used to calculate the spatial correlation of genome-wide interval datasets. The permutation tests with 100 times were performed to verify whether TEs enriched in the regulatory elements or not.

### Identification of accessible TEs

As previously reported [52], accessible TEs were identified by the overlapping between TEs and peaks, and the evaluation criterion is that at least 50% of the overlap should be TE while at least 20% of the overlap should be peak region. Accessible TEs that were only identified in one tissue was defined as tissue-specific accessible TEs while accessible TEs that were identified in all tissues was defined as shared accessible TEs. GO enrichment analysis of tissue-specific accessible TEs and shared accessible TEs were performed using ClueGO [46].

### Multiple sequence alignment

Virus sequences were obtained from a study (unpublished) by self-assembly and comparison with the database [53]. The conserved accessible TEs sequences and viral sequences were put together. Then, we used MAFFT v7.407 [54] with the automatically detected parameters (--auto) to identified sequence homology. FastTree v2.1.11 [55] was used to infer approximately-maximum-likelihood phylogenetic trees from alignments of nucleotide sequences. Finally, experiment visualization features in FigTree v1.4.4 [56].

### The enrichment of GWAS signals

The pig GWAS data of fatty acid was collected from a published study [57]. A total of 191 significant GWAS loci were obtained. Then, the enrichment analysis of these loci was performed with regulatory elements.

## Results

### Mapping the open chromatin states of 20 pig tissues

To map genome-wide regulatory elements in pig, we performed ATAC-seq and RNA-seq on 40 samples representing 20 tissues, namely, brain, cerebellum, hypothalamus, medulla spinalis, lung, bronchia, parotid gland, pharynx, stomach, liver, SI, kidney, ovary, cervix, heart, thymus, *longissimus* muscle (LD), semimembranosus muscle (SM), backfat and leaf fat from two female DLY pigs at 6 months (Fig. 1a), representing two biological replicates per tissue. The data was subjected to analysis following the workflow shown in Fig. 1b.

**Fig. 2:**
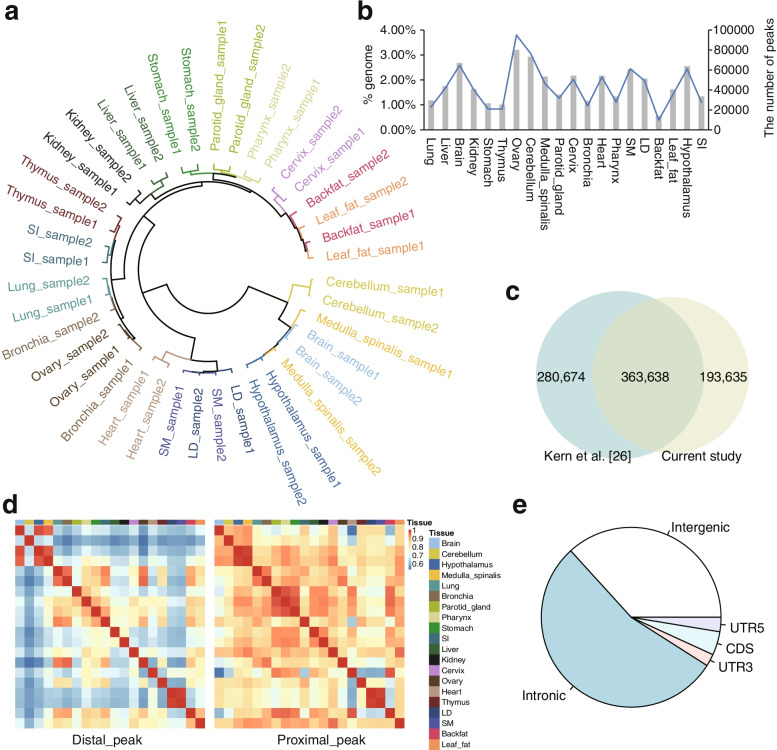
Identifying the landscape of chromatin accessibility via ATAC-seq in pigs. **a** Hierarchical clustering of samples using ATAC-seq signal intensity. **b** Distribution of the peak number and genome coverage in each tissue. **c** Venn plot showing the overlap of peaks identified in this study with those reported in Kern et al. [26]. **d** Pearson correlation heatmaps of 20 tissues by density of ATAC-seq distal peaks (left) and ATAC-seq proximal peaks (right). **e** Pie chart represents the proportion of peaks located in introns, intergenic, 5′UTR, 3′UTR and CDS regions

After quality control, we obtained a total of 2.43G uniquely mapped reads from the 40 samples (Additional file 1: Table S1). The reads showed enrichment around TSSs and a periodical fragment size distribution corresponding to the nucleosome-free regions (NFR) (< 100 bp) and mono-, di-, and tri-nucleosomes (~ 200, 400 and 600 bp, respectively) (Additional file 2: Fig. S1a, S1b). Furthermore, we computed quality related statistics FRIP (fraction of reads in peaks), TSS score, library complexity (NRF (Non-redundancy Fraction), PBC1 and PBC2 (PCR Bottlenecking Coefficient 1 and 2)) and cross correlation (NSC and RSC) (Additional file 1: Table S1). These statistics suggested that the data generated hereby are of good quality. We identified an average of 77,096 peaks with average size of 448 bp per tissue (Additional file 2: Fig. S1d). Peaks from all samples were merged into 557,273 non-redundant peaks, among which 363,638 (65.25%) overlapped with peaks identified in Kern et al. [26] (Fig. 2c), while the rest of peaks were newly identified owing to more tissue types were profiled in this study.

To obtain a set of peaks with higher confidence, we only kept peaks that were evidenced in both biological replicates for each tissue, and finally retained 267,836 merged peaks for further analysis. Most of the 267,836 peaks located in intronic (54.23%) and intergenic (36.75%) regions, meanwhile, 4.23%, 2.59% and 2.20% peaks were evidenced in CDS, 5′UTR and 3′UTR regions (Fig. 2e). Similar to the results obtained in the previous study [25], we found that accessible chromatin regions vary greatly across tissues (range: 9729 to 94,946), covering 0.56% to 3.21% sequences of the reference genome (Fig. 2b), with a good overlap with those reported in independent studies by tissues (Additional file 1: Table S4).

Hierarchical clustering based on the peak intensity clearly separated different tissue types (Fig. 2a), indicating that the peak intensities largely reflect tissue identity. We grouped the 267,836 peaks into 20,488 proximal peaks (within 1 Kb of TSS) and 247,248 distal peaks (away from TSS 1 Kb). As expected, the distal peaks displayed stronger tissue specificity comparing to proximal peaks (Fig. 2d and Additional file 2: Fig. S1c), agreeing with the reported characteristics of promoters and enhancers [58].

To investigate the effect of regulatory elements on gene expression, RNA-seq was successfully performed on 38 out of the 40 ATAC-Seq samples. The summary statistics of each RNA library is listed in Supplementary Table 2. A total of 17,624 genes were identified. The reproducibility of the 38 RNA-seq data was verified by hierarchical clustering of gene expression (Additional file 2: Fig. S2a).

### Tissue specificity of the open chromatin peaks

**Fig. 3:**
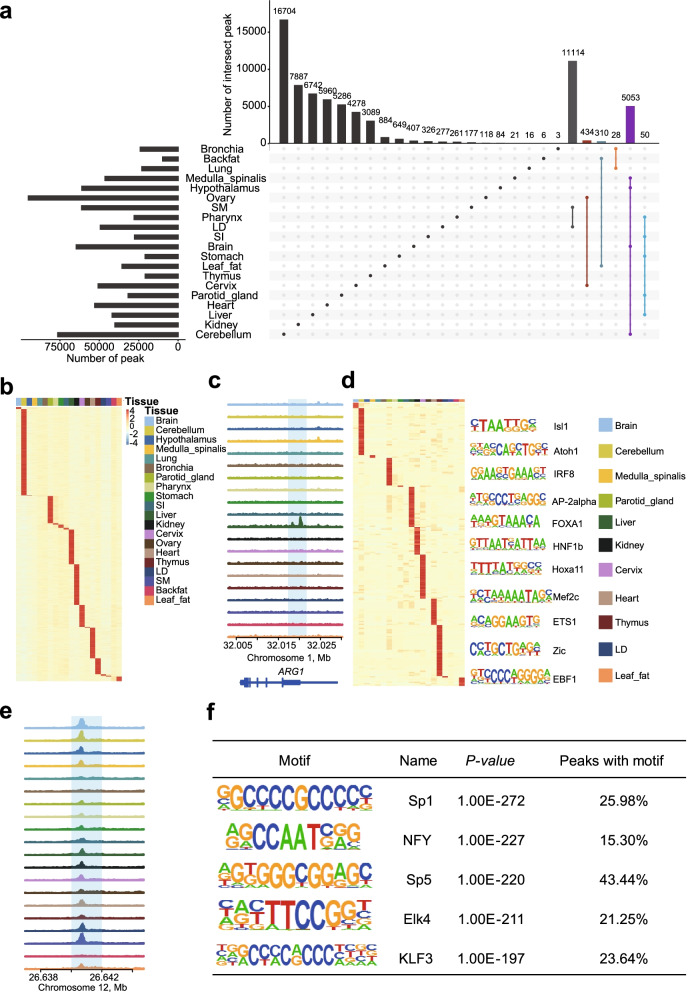
Functional annotation of system-specific peaks. **a** Intervene plot showing the number of system-specific peaks and tissue-specific peaks. **b** Heatmap of tissue-specific peaks in 20 tissues. Each row of the heatmap shows normalized density of read-depth at one peak**. c** Representative examples of liver specific peaks which located close to ARG1(A protein coding gene expressed primarily in the liver and involved in the urea cycle). **d** Enrichment of tissue specific transcription factor motifs in each tissue (left). The columns represent 20 tissues. The rows represent motifs. The *P*-values were generated by HOMER. Representative examples of tissue specific transcription factor motifs were displayed (right). **e** Representative examples of tissue conserved peak. **f** Top 5 transcription factors with binding motifs enriched in tissue conserved peaks

To explore the key regulatory elements that determine specialized functions of tissues, we defined peak whose average intensity was at least two folds in the target tissue relative to any other tissues as tissue-specific peak, and identified 16,704, 7887, 6742, 5960, 5286, 4278, 3089, 884, 649, 407, 326, 277, 261, 177, 118, 84, 21, 16, 6 and 3 tissue-specific peaks in cerebellum, kidney, liver, heart, parotid gland, cervix, thymus, leaf fat, stomach, brain, SI, LD, pharynx, SM, ovary, hypothalamus, medulla spinalis, lung, backfat and bronchia, respectively (Fig. 3a, b). We exemplified a liver specific peak located close to *ARG1* (Fig. 3c and Additional file 2: S2e), a protein coding gene expressed primarily in the liver and involved in the urea cycle [59]. The genes locate closest to the tissue-specific peaks were enriched in tissue-specific pathways, such as the heart and circulatory system development for heart specific peaks (Additional file 2: Fig. S2f), such as heart development and circulatory system development in heart. Given that the numbers of tissue-specific peaks were largely affected by the inclusion of physiologically similar tissues, we further determined peaks that specific to a certain tissue system using a similar rule applied on the individual tissues, and identified 11,114, 434, 310, 28, 5053 and 50 specific peaks for locomotor system, reproductive system, endocrine system, respiratory system, nervous system and digestive system, respectively, located close to genes with functions matching the biology of corresponding tissues (Fig. 3a). For example, genes expressed specifically in nervous system were significantly involved in synaptic membrane (*q*-value = 1.39E-32), while those expressed specifically in locomotor system were significantly associated with muscle structure development (*q*-value = 5.68E-28) (Additional file 2: Fig. S2d).

Next, we investigated enrichment of TF binding motifs in the tissue-specific peaks using HOMER [47]. We defined the TF motifs with enrichment strength (−log (*P*-value)) in one tissue were at least two folds relative to the other tissues as tissue-specific, and identified 205 tissue-specific TF motifs (Fig. 3d). These included motif of ATOH1 which controls primary cilia formation that facilitates SHH-Triggered granule neuron progenitor proliferation [60] was evidenced to be cerebellum specific, and motif of MEF2C which controls cardiac morphogenesis [61] was enriched in heart.

### Ubiquitously accessible chromatin peaks

Meanwhile, we also identified 8734 peaks that consistently active in at least 90% of investigated tissues (Fig. 3e). The genes locate nearest to the conserved peaks were enriched for pathways like cytoskeleton and mitotic cell cycle that have housekeeping functions (Additional file 2: Fig. S2d). The top 5 enriched TF motifs (SP1, NFY, SP5, ELK4 and KLF3) (Additional file 1: Table S5) participate in chromatin remodeling and transcriptional activation (Fig. 3f), which are indispensable transcription factors in growth and development [62].

### Tissue-specific gene expressions

We applied similar approach to investigate tissue specificity of gene expressions, and identified 135, 528, 439, 711, 228, 181, 117, 467, 40, 75, 469, 486, 94, 581, 124, 117, 411, 34, 336 and 1590 tissue-specific genes that were found to be specific in backfat, brain, bronchia, cerebellum, cervix, heart, hypothalamus, kidney, LD, leaf fat, liver, lung, medulla spinalis, ovary, parotid gland, pharynx, SI, SM, stomach and thymus, respectively (Additional file 2: Fig. S2b). For example, *MYH7*, a gene described to be responsible for hypertrophic cardiomyopathy, was highly expressed in heart than in other tissues (Additional file 2: Fig. S2c). The GO analysis showed these tissue-specific genes were associated with tissue-specific biological processes (Additional file 2: Fig. S2b). For example, genes significantly expressed in cerebellum were related to the function of synaptic signaling, and genes significantly expressed in thymus were related to the function of T cell activation (Additional file 2: Fig. S2b).

### Joint profiling of chromatin accessibility and gene expression

**Fig. 4:**
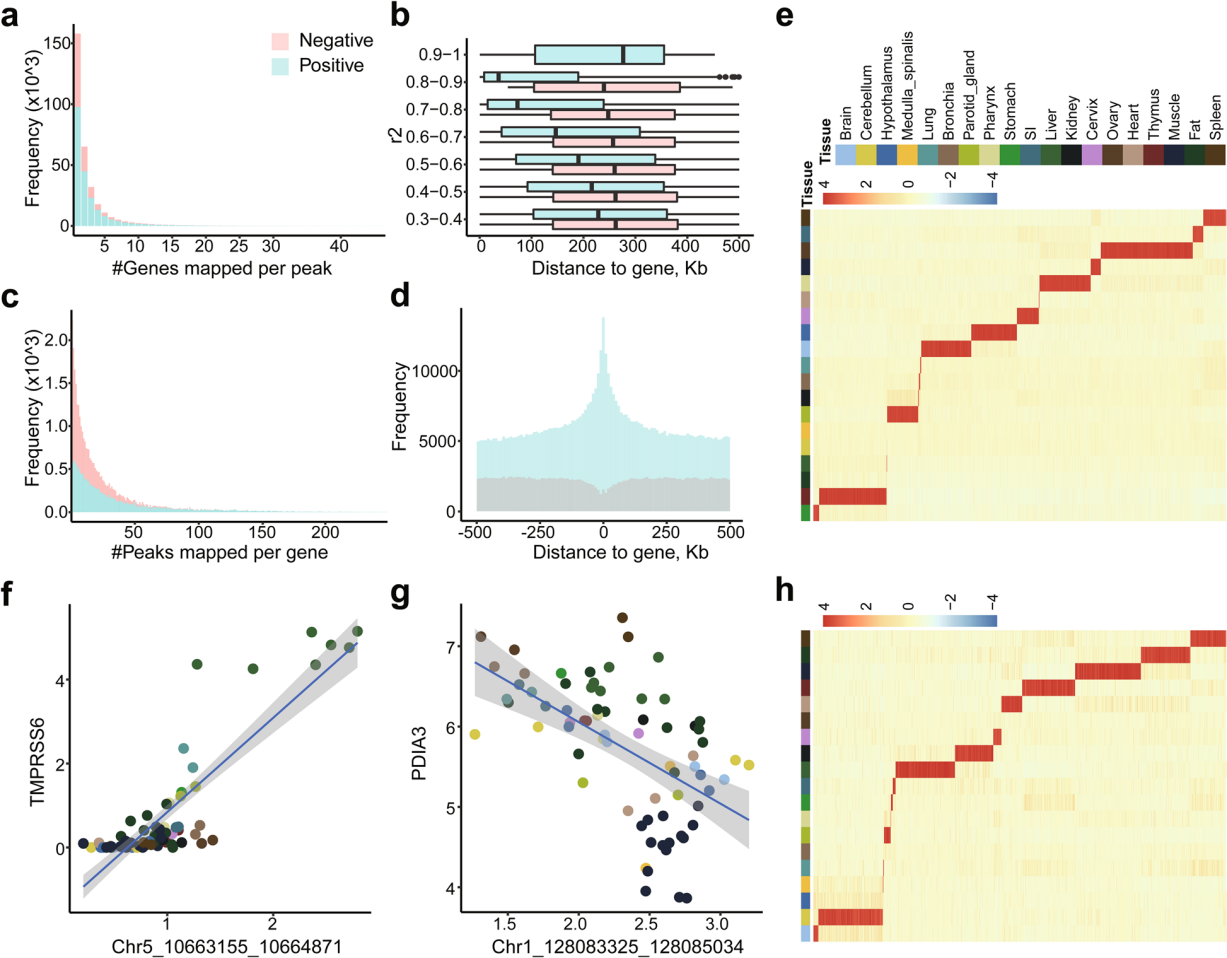
Characterizing the correlations between peak intensity and gene expression of nearby genes. **a** and **c** The number of genes regulated by one peak, and the number of peaks regulated one gene. The X-axis of the histogram represents different groups that were classified according to the associated gene (peak) numbers per peak (gene), and the Y-axis represents the gene (peak) count for each group. Positive peak - gene pairs and negative peak - gene pairs are distinguished by different colors. **b** Bar plot of the relationship between the correlation and distance of peak - gene pairs. **d** Distribution of the distance for significant positive peak - gene pairs and negative peak - gene pairs. **e** and **h** Heatmap of tissue-specific peaks (genes) from all significant peak - gene pairs. **f** Representative examples of a significant tissue-specific peak - gene pair which peak and gene are both tissue-specific. The dots represent peak intensity and gene expression from each sample. Different colors indicate different tissue types. **g** An representative examples of a significant conserved peak - gene pair. The peak and gene are both expressed ubiquitously across tissues. The dots represent peak intensity and gene expression from each sample. Different colors indicate different tissue types

To investigate the regulatory elements that link to gene transcription, we examined correlations of gene expressions with intensities of ATAC peaks within 500 Kb from the TSSs of corresponding genes, as majority of promoter-anchored chromatin interactions were within 500 Kb [63, 64]. The analysis was performed on the integrated dataset of 527,718 peaks and 17,634 genes from 71 samples, including 33 samples from public datasets (Additional file 1: Table S3, Additional file 2: Fig. S3). We identified a total of 827,942 significant peak - gene associations (*q*-value < 0.01) that involves 255,166 peaks and 17,493 genes (Additional file 2: Fig. S4). Majority of the significant correlations (406,896/827,942, 49.15%) are positive only, while we also identified 10.54% negative correlations only. For example, a peak (chr5:10,663,155-10,664,871) has high intensity in liver showed positive correlation with *TMPRSS6* (Spearman *r*^2^ = 0.77, *q*-value = 6.43E-12) (Fig. 4f), which encodes a type II transmembrane serine protease exclusively produced by liver [65]. We also exemplified negative peak - gene correlation where a peak (chr1:128,083,325-128,085,034) show strong negative correlation with expression levels of *PDIA3* (Spearman *r*^2^ = − 0.60, *q*-value = 1.42E-6) (Fig. 4g). In addition, there are 40.31% peak - gene correlations showed both positive and negative correlations, indicating that the identity of regulatory elements was mutable.

On average, there were 2.87 genes per peak and 35.86 peaks per gene in positive correlations while 2.13 genes per peak and 14.49 peaks per gene in negative correlations (Fig. 4a, c). Interestingly, we observed that the strength of positive and negative correlations showed distinct distribution against peak - gene distances (Fig. 4b). The positive correlations were enriched around the TSSs of genes, while the negative correlations appeared to be underrepresented in TSS regions (Fig. 4d). We identified 1786 tissue-specific genes were associated with 38,574 tissue-specific peaks within 500 Kb from the TSS regions (Fig. 4e, h and Additional file 2: Fig. S4), suggesting important roles of tissue-specific chromatin accessibility in driving the tissue-specific genes expression thereby encoded tissue-specific biological functions.

### Open chromatin states of transposable elements in different pig tissues

Over 40% of the sequences in the non-coding regions of the mammalian genome are TEs whose functions were largely unexplored. To understand the functions of TEs in pig tissues, we examined the chromatin accessibility of genome wide TEs. We found 26.91% of the 267,836 open chromatin regions were overlapped with at least one TE.

**Fig. 5:**
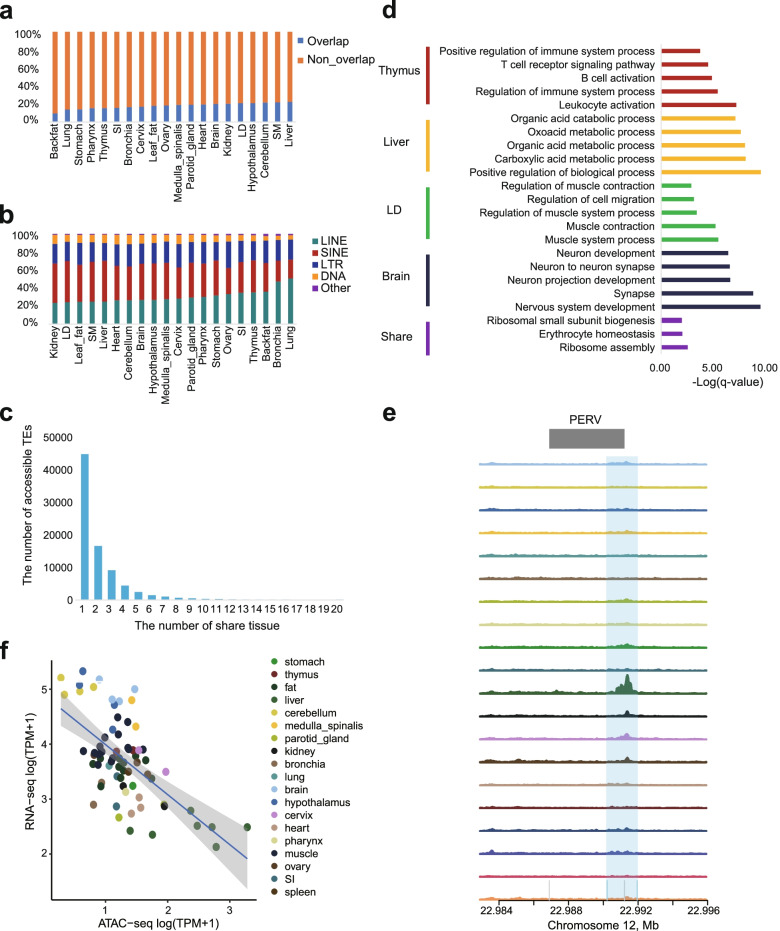
Accessible transposable elements (TEs) in 20 pig tissues. **a** Distribution of ATAC-seq peaks identified in 20 pig tissues associated with TEs. **b** TE class distribution of accessible TEs in 20 pig tissues. **c** Distribution of accessible TEs among 20 pig tissues. **d** Functional enrichment of genes for 4 tissue-specific and tissue-conserved accessible TEs based on Gene Ontology (GO) biological processes. **e** Comparison the tracks of peak activity across all tissues; Y-axis corresponding to the normalized read depths of ATAC-seq data. **f** Scatter plot showing the correlation between the intensity of peak at chr12:22,990,219-22,991,950 and the expression of *PIP4K2B* gene

The spatial correlation analysis between TEs and open chromatin regions calculated by GenometriCorr [51] suggested underrepresentation of ratio of TEs located in the open chromatin regions than random expectation, indicating that TEs are tend to be epigenetically silenced. Despite this, each tissue contains an average of 17.96% peaks that were overlapped with at least one TE, range from 9.49% to 22.33% (Fig. 5a). The SINE, LINE and LTR accounted for approximately 40%, 30% and 23% of the accessible TEs, respectively, across the 20 tissues (Fig. 5b). We found over 50% of these TEs existed in a single tissue were located close to genes involved in tissue-specific functions (Fig. 5c, d). For example, we observed enrichment of T cell receptor signaling pathway enriched in thymus while muscle system process enriched in LD (Fig. 5d). These results suggested that accessible TEs existed in one tissue exhibit strong tissue-specific regulatory roles, which was consistent with the previous reports [66, 67]. Meanwhile, we observed approximate 0.19% of accessible TEs were commonly accessible in all 20 tissues (Fig. 5c). The conserved accessible TEs were more associated with housekeeping functions (e.g., ribosome assembly) (Fig. 5d).

Mounting evidence suggests that specific TEs in pigs, especially PERV, could potentially lead to immunodeficiency and tumorigenesis, making it difficult for xenotransplantation [68, 69]. Upon further inspection, we found a total of 10 unique PERVs overlapped with 13 peaks (Additional file 1: Table S6). For example, the PERV (sPERV-JX2like-12-23.0 M) was overlapped with the peak (chr12:22,990,219-22,991,950) which showed high intensity in liver (Fig. 5e), significantly correlated with multiple genes (*NEUROD2*, *ENSSSCG00000017507*, *CACNB1*, *PLXDC1*, *PIP4K2B*). Among them, *PIP4K2B*, which participates in 1-phosphatidylinositol-4-phosphate 5-kinase activity and IP6 [70], the product of 1-phosphatidylinositol-4-phosphate 5-kinase, can facilitates assembly of the immature HIV-1 Gag lattice [71], shows highly correlation (Fig. 5f), suggesting potential role of the PERVs in facilitating virus infection.

Besides this, we also focused on the functions of other genes regulated by PERVs. For example, *GTPBP8* [72], *BCKDK* [73], *IDH3B* [74] and *IDH1* [75] were related to the function of mitochondrion; *CD2BP2* [76] and *FUS* [77] involved in mRNA splicing; *BOC* [78] and *FZD5* [79] participated in signal transduction pathway. Taken together, these results suggested that PERV which act as regulatory element involved in a variety of biological functions.

**Fig. 6:**
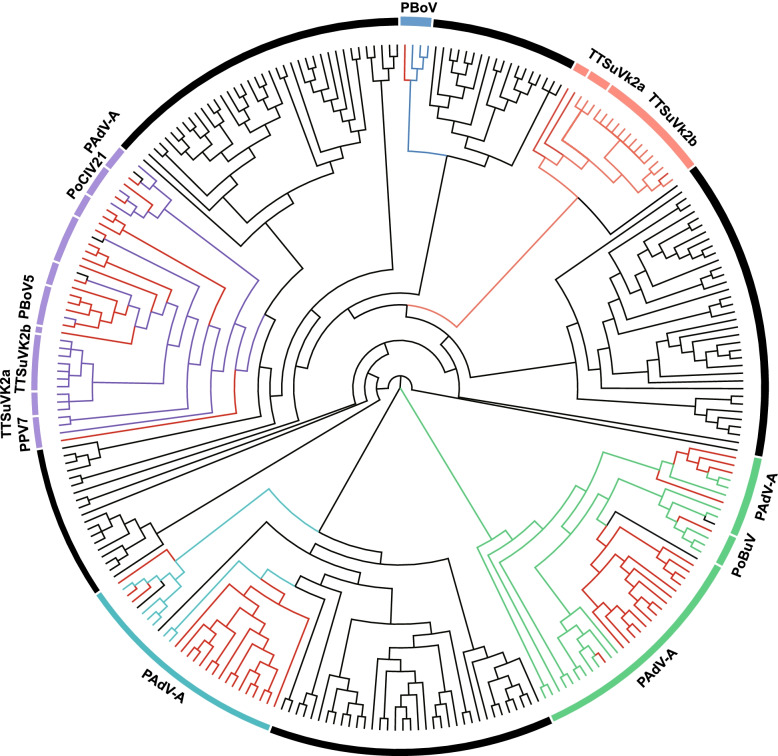
Phylogram of 124 viruses and 162 accessible TEs sequences. Color-highlighted clusters indicate pig virus sequences. PBoV: porcine bocavirus; TTSuVK2a(b): Torque teno sus virus k2a(b); PADV-A: porcine mastadenovirus; PoBuV: Protoparvovirus Zsana/2013/HUN; PPV7: Porcine parvovirus 7; PoCIV21: Po-Circo-like virus 21. The red branches mark peaks that clustered with viruses

In this study, we identified a total of 162 TEs that are accessible in all investigated tissues. To trace the origin or evolutionary history of these ubiquitously accessible TEs, we calculated sequence similarity between 124 viral sequences (Additional file 1: Table S8) and 162 conserved accessible TEs sequences, and constructed a phylogenetic tree to reconstruct evolutionary history. Interestingly, we observed some conserved accessible TEs sequences were grouped with some viruses whose host was pig (Fig. 6), such as PBoV, TTSuVK2a, TTSuVK2b, PADV-A, PoBuV, PPV7 and Po-Circo-Like Virus 21 (Additional file 1: Table S9). We performed GO and KEGG enrichment analysis of all genes associated with conserved accessible TEs, and found that the biologic process of mitochondrial depolarization was highly enriched (Additional file 1: Table S10), which indicated that accessible TEs derived from viral sequences have a potential regulatory function in clearance of excess or impaired mitochondrial. Altogether, these evidences suggested that some special viral sequences have been integrated into the host genome and retained as regulatory elements that could play constitutive regulatory functions.

### An annotation resource for regulatory mechanisms in complex traits

**Fig. 7:**
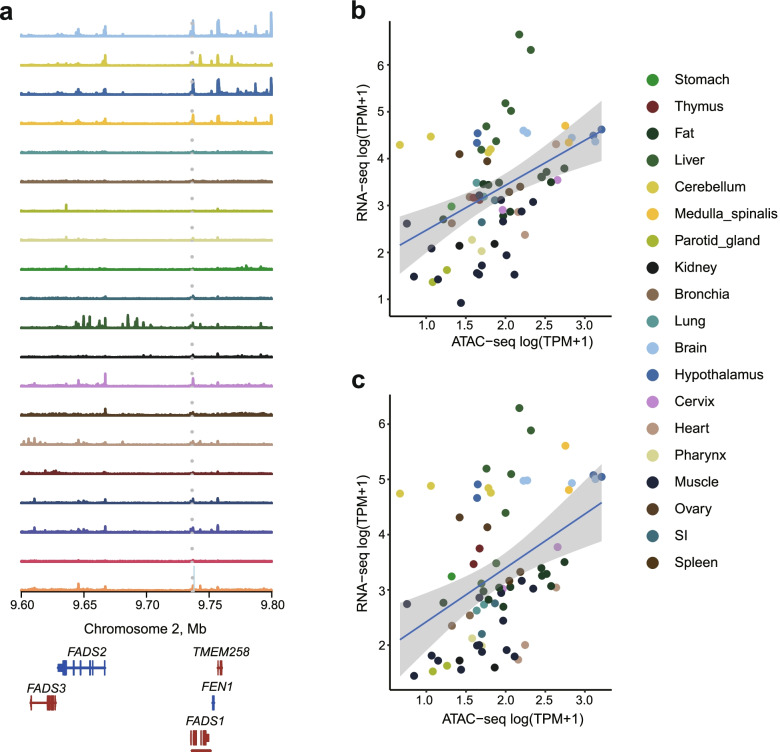
Overlap with GWAS SNPs. **a** The GWAS signal for fatty acid located within a peak in intergenic region. **b** Scatter plot showing the correlation between the peak at chr2:9,736,357-9,737,907 with expression of *FADS1* gene. **c** Scatter plot showing the correlation between the peak at chr2:9,736,357-9,737,907 with expression of *FADS2* gene

To demonstrate the utility of such resource in annotating candidate casual variants for complex traits, we examined overlap of 191 significant variants for fatty acid composition identified in a genome sequence based imputation GWAS [57] with the ATAC-seq peak regions identified in this study. A total of 22 variants were found to be coincident with the ATAC-seq peaks (Additional file 1: Table S7). Among these, a lead SNP (chr2:9,736,686, *P* = 2.10E-10) for C20:3n-6/C18:2n-6 was located in an ATAC-seq peak (chr2:9,736,357-9,737,907) that showed high intensity in brain and moderate intensity in muscle (Fig. 7a). This peak showed significant correlation with three genes (*FADS1*, *FADS2* and *RAB3IL1*) (Additional file 1: Table S7). Among them, *FADS1* (*q* = 1.56E-4) which exhibited strongest correlation (Fig. 7b), encodes delta-5 desaturase, one of the rate-limiting enzymes in the endogenous synthesis of polyunsaturated fatty acids (PUFAs) [80]. *FADS2* which converts linoleate and alpha-linolenate into PUFAs, is one of the key limiting enzymes in the lipid metabolic pathway [81] (Fig. 7c). In addition, several other genes related to fatty acid have been identified. Osteoglycin (*OGN*) is involved in matrix assembly, cellular growth, and migration. Previous study showed that *OGN* was associated with fat acids composition traits to some extent [82] and regulated lipid differentiation through the Wnt/β-catenin signaling pathway [83]. *TEAD3* which participates in the fatty acid, triacylglycerol, and ketone body metabolism pathway from the Reactome Pathways database [84], is a member of the transcriptional enhancer factor (TEF) family of transcription factors. Further experiment is needed to validate effect of the variants on the accessibility of the peaks, and in turn the expression of relevant genes and fatty acid composition phenotypes.

## Discussion

Regulatory elements are crucial for gene expression regulation, which involved in the initiation and regulation of transcription in different tissues and organs and provided precise control of genes and restrict gene expression to certain cells and tissues throughout life [85]. Activation of regulatory elements needs ‘pioneer’ factor opening the compacted chromatin to recruit transcription coactivators and chromatin remodeling proteins [86, 87]. Regulatory elements usually contain multiple transcription factors binding sites (TFBS) which recognized by TFs. Genetic variations in regulatory elements affect gene expression usually through disrupting TFBS [88]. Therefore, the generation of multi-tissues atlas of open chromatin enabled exploration of gene expression regulation and non-coding genetic variations.

In this study, we performed ATAC-seq and RNA-seq across 20 tissues in pig at the same development stage to characterized chromatin state landscape, uncovering extensive tissue-specific regulation of gene expression. However, not a single study until now, as far as we are aware, has comprehensively and systematically investigated chromatin accessibility in multi-tissues by ATAC-seq and conjunction with transcriptomic analysis. In our study, we observed that the patterns of TSS proximal regulatory elements were more similar across tissues while distal regulatory elements were more tissue-specific, reflecting the architecture and function of distal regulatory elements have strong tissue specificity [89].

Consistent with the previous reports that regulatory elements mediate specific differentiation of tissues or cells [90, 91], we identified a large number of tissue-specific open chromatin regions which regulate the genes with function matching the biology of corresponding tissues. In addition, we identified tissue-specific TF motifs based on the enrichment of their cognate binding motifs, such as ATOH1 enriched in cerebellum and FOXA1 enriched in liver. And we acknowledge that TF motifs enrichment analysis needs to verify using other approaches such as ChIP-seq for specific TFs.

Connecting peaks to their target genes remains challenge given that Hi-C data is not available for the samples under study, particularly for distal peaks which are far from target genes, and regarding genes nearest to peaks as targets is error prone [92–94]. To solve this problem, we combine other published data to reveal the association between peak intensity and gene expression, which has been proved effective [90]. We observed significant negative peak - gene pairs showed strikingly different peak - gene distances compared to that in positive correlation pairs, which suggested that positive and negative correlation pairs may have different regulatory mechanism. To further understand tissue-specific regulatory relationships between peak intensity and gene expression, we filtered for tissue-specific correlation pairs (Additional file 2: Fig. S4). Our results suggest tissue-specific genes expression may occur through the activation of tissue-specific regulatory elements. Whereas, the above predictions require further support using 3D chromosome conformation data or validated through CRISPRi experiments.

To deepen our understanding of regulatory elements, here we performed profiles in TEs as they can provide binding sites for TFs and behave as alternative promoters and enhancers [95]. In this study, we observed a small proportion of accessible TEs, which were in good accordance with the classical polarization theory that TEs promote genetic innovation but also threaten genome stability [96]. Similar to previous study [52], we observed most of accessible TEs only present in one tissue. And tissue-specific accessible TEs were enriched in tissue-specific biological pathways, which support the findings of previous studies [52, 97–99]. Endogenous retroviruses (ERVs) are LTR retrotransposons (class I of TEs), which originated from exogenous retroviruses that infected the germ line throughout evolution [100, 101], which could manifest their function across multiple organs in an animal, thereby could have fundamental functional impact. In order to trace the potential source of accessible TEs, we performed multiple sequence alignments between the viral sequences and the conserved accessible TEs sequences, and found some candidate viral sequences (PBoV, TTSuVK2a, TTSuVK2b, PADV-A, PoBuV, PPV7 and Po-Circo-Like Virus 21) might be the source of conserved accessible TEs.

The annotation of regulatory elements has proven highly effective for the identification of candidate causative variants and screening candidate genes for complex traits [88, 102, 103]. Similarly, our results demonstrated that variants of fatty acid were enriched in active regulatory elements annotated by this study. Specifically, we speculate that a potential causative SNP (chr2:9,736,686, *P* = 2.10E-10) that was associated with C20:3n-6/C18:2n-6 and found within a tissue-conserved promoter, may regulate the expression of *FADS1* which encodes the Δ5 desaturase enzyme - one of the rate-limiting enzymes in the endogenous synthesis of polyunsaturated fatty acids (PUFAs) [104]. Taken together, our data unveiled the chromatin landscapes for studying the regulation of gene expression, provided potential sources of chromatin accessibility and deconstructed underlying mechanisms of complex traits in pig. Further investigations with additional complementary data—such as single cell and different development stage data—are warranted to fully dissect biological mechanisms of gene expression.

## Conclusions

We performed an integrated analysis of transcriptome sequencing and transposase-accessible chromatin with high throughput sequencing and provided a comprehensive understanding of tissue-specific accessible chromatin regions and tissue-specific TF motifs. Then, we constructed the relationship between regulatory elements and their target genes. This large regulatory network will serve as a foundation for understanding the precise regulatory mechanisms in different tissues of pig. We also analyzed accessible TEs which act as candidate regulatory elements. In addition, we analyzed the sequence characteristics of regulatory elements, and found some virus sequences similar to regulatory element sequences, which provided a certain basis for the essential characteristics of regulatory elements. Finally, the regulatory elements identified in this paper were used to annotate the GWAS loci that affect fatty acid traits, providing a reference for genetic analysis of complex traits.

## Supplementary Information

**Additional file 1: Table S1.** Summary information on the ATAC-seq samples investigated in this study. **Table S2.** Summary information on the RNA-seq samples investigated in this study. **Table S3.** The information of download data. **Table S4.** Comparison with peaks of previous study designed by zhou et al. **Table S5.** Motif enrichment analysis of shared peaks. **Table S6.** The information of PERV overlapped peaks. **Table S7.** The information of GWAS SNPs which located in peak regions. **Table S8.** The information of virus data. **Table S9.** The information of virus data with sequence homology to shared open TEs. **Table S10.** GO and KEGG enrichment analysis of conserved accessible TEs homologous to viral sequences.

**Additional file 2: Fig. S1.** The analysis process and characteristics of peaks. (a) and (b) The ATAC-seq signal enrichment around TSSs and insert size distribution of ATAC-seq for 2 samples (brain_sample1 and brain_sample2). (c) Proportion of tissue-specific peaks that were distal peaks or proximal peaks. (d) Distribution of peak size obtained in this study. **Fig. S2.** Identifying the functional pathway and tissue specificity via RNA-seq. (a) Hierarchical clustering using gene expression. (b) Functional enrichment of tissue-specific expression genes for each tissue based on Gene Ontology (GO) biological processes (left). Distribution of the number of tissue-specific genes (right). (c) Representative examples of heart specific gene which is highly expressed in heart tissue. (d) Bar plot showing representative biological processes enriched in target genes of the system specific peaks and conserved peaks. Different colors refer to different systems, and light yellow indicates biological processes enriched in target genes of conserved peaks. (e) Bar plot showing peak intensity of liver specific peaks which located close to *ARG1* (a protein coding gene expressed primarily in the liver and involved in the urea cycle). (f) Functional enrichment of genes for each tissue based on GO biological processes. The columns represent 20 tissues. The rows represent GO terms in each tissue (upper). Representative examples of enriched biological processes in target genes of heart specific peaks (bottom). **Fig. S3.** Hierarchical clustering inferred from peak intensity and gene expression. (a) Hierarchical clustering using peak intensity. The top 100,000 peaks with high intensity were used for the analysis. (b) Hierarchical clustering using gene expression. The top 5000 highly variable genes were used for the analysis. **Fig. S4.** The flowchart and outcomes from the correlation between peak intensity and gene expression.

## Data Availability

Supporting data produced in this study have been uploaded to National Genomics Data Center with the accession number CRA007240.

## Abbreviations

TF: Transcription factor
ATAC-seq: Transposase-accessible chromatin with high throughput sequencing
DNase-seq: DNase I hypersensitive site sequencing
ERVs: Endogenous retroviruses
FRIP: Fraction of reads in peaks
LD: *Longissimus* muscle
MNase-seq: Micrococcal nuclease sequencing
NFR: Nucleosome-free regions
NSC: Normalized strand cross-correlation coefficient
OGN: Osteoglycin
PADV-A: Porcine mastadenovirus A
NRF: Non-redundancy Fraction
PBC1: PCR Bottlenecking Coefficient 1
PBC2: PCR Bottlenecking Coefficient 2
PBoV: Parvoviridae Bocaparvovirus Porcine bocavirus
PoBuV: Protoparvovirus Zsana/2013/HUN
PoClv21: Po-Circo-Like Virus 21
PPV7: Porcine parvovirus 7
PUFAs: Polyunsaturated fatty acids
RSC: Relative strand cross-correlation coefficient
SI: Small intestine
SM: Semimembranosus
TE: Transposable element
TEF: Transcriptional enhancer factor
TFBS: Transcription factors binding site
TPM: Transcripts per million
TSS: Transcription start site
TTSuVK2a: Torque teno sus virus k2a
TTSuVK2b: Torque teno sus virus k2b
