# Supplementary figures and images for: Construction of a transposase accessible chromatin landscape reveals chromatin state of repeat elements and potential causal variant for complex traits in pigs
Source: J Anim Sci Biotechnol. 2022 Oct 11;13:112. doi: 10.1186/s40104-022-00767-3 (PMC9552403; doi:10.1186/s40104-022-00767-3)

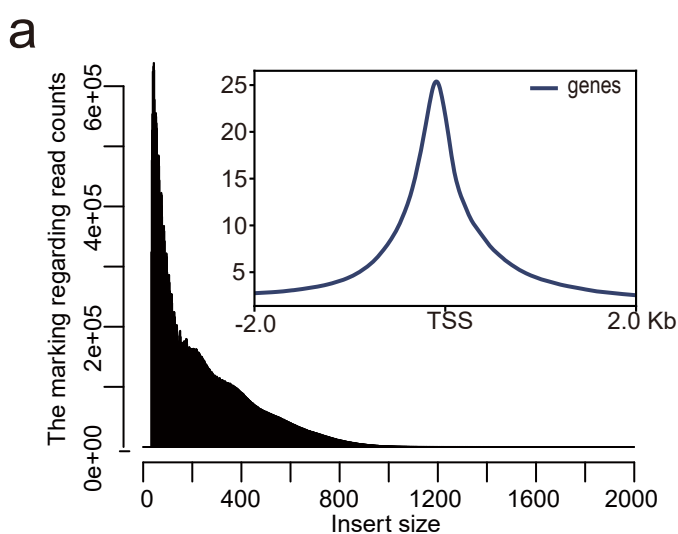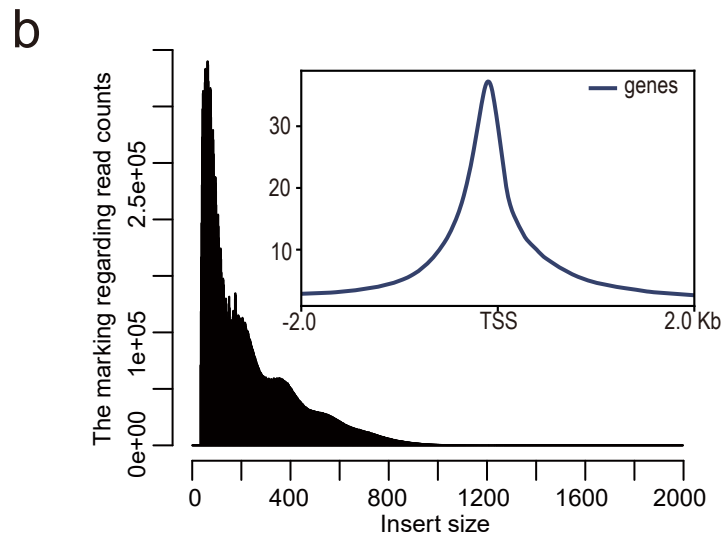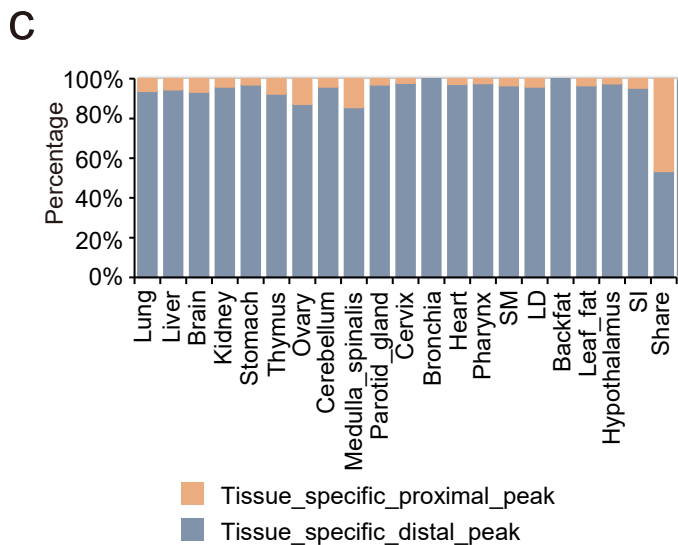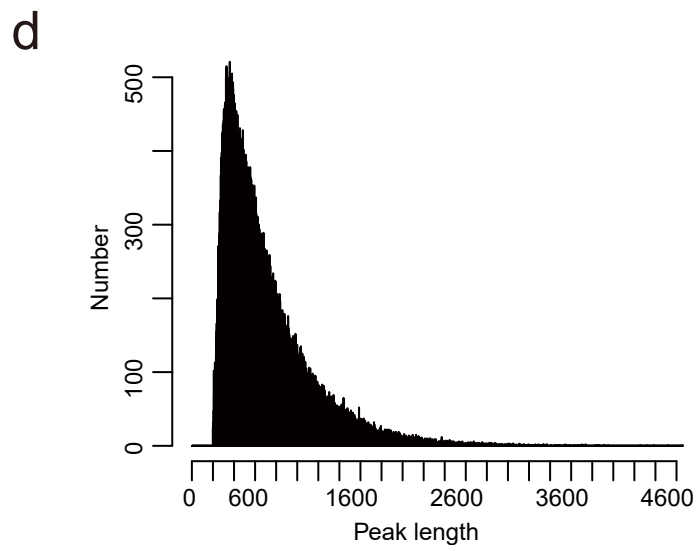

Supplement: Supplementary file 1 — Additional file 1: Table S1. Summary information on the ATAC-seq samples investigated in this study. Table S2. Summary information on the RNA-seq samples investigated in this study. Table S3. The information of download data. Table S4. Comparison with peaks of previous study designed by zhou et al. Table S5. Motif enrichment analysis of shared peaks. Table S6. The information of PERV overlapped peaks. Table S7. The information of GWAS SNPs which located in peak regions. Table S8. The information of virus data. Table S9. The information of virus data with sequence homology to shared open TEs. Table S10. GO and KEGG enrichment analysis of conserved accessible TEs homologous to viral sequences. [file 40104_2022_767_MOESM1_ESM.zip › Supplemental figure01.pdf]

**a**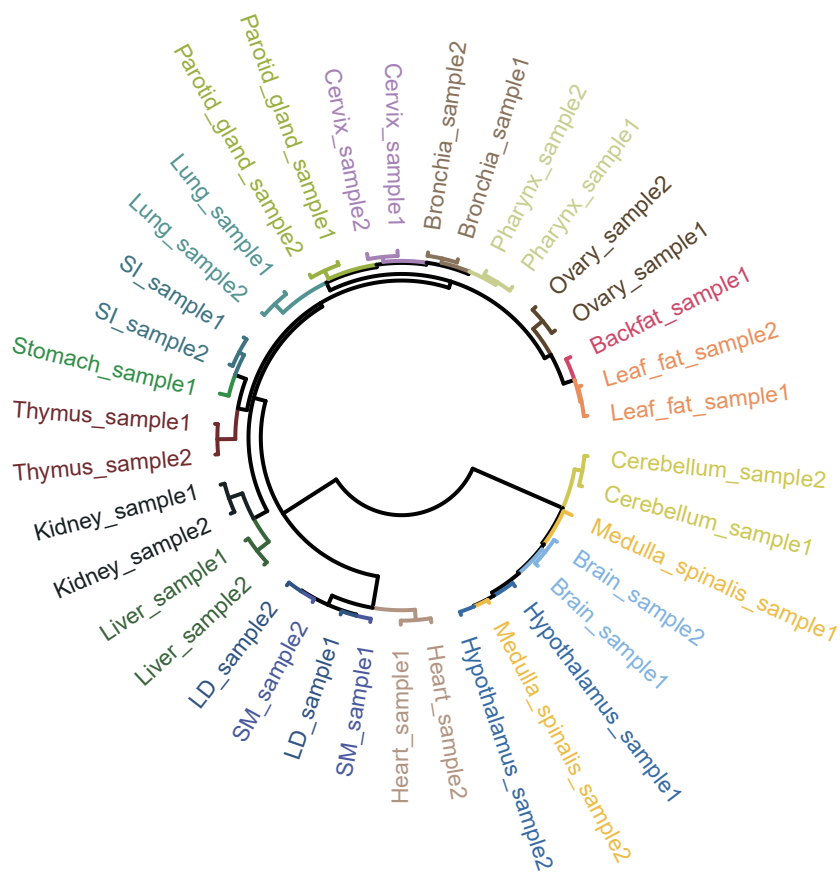**b**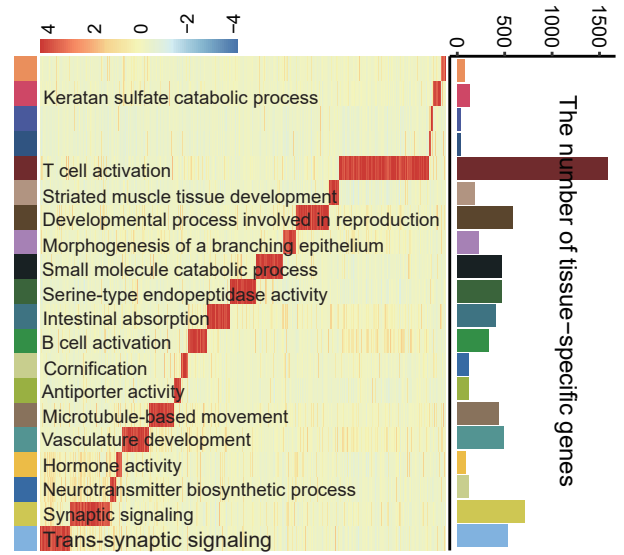**c**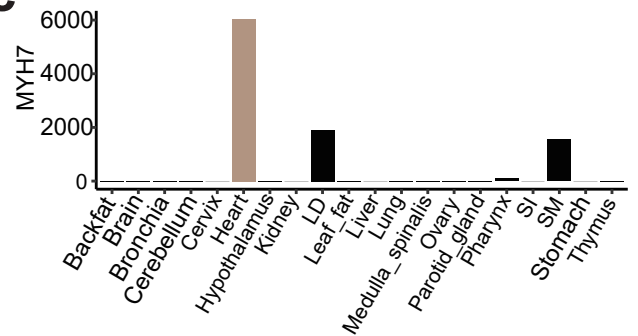**d**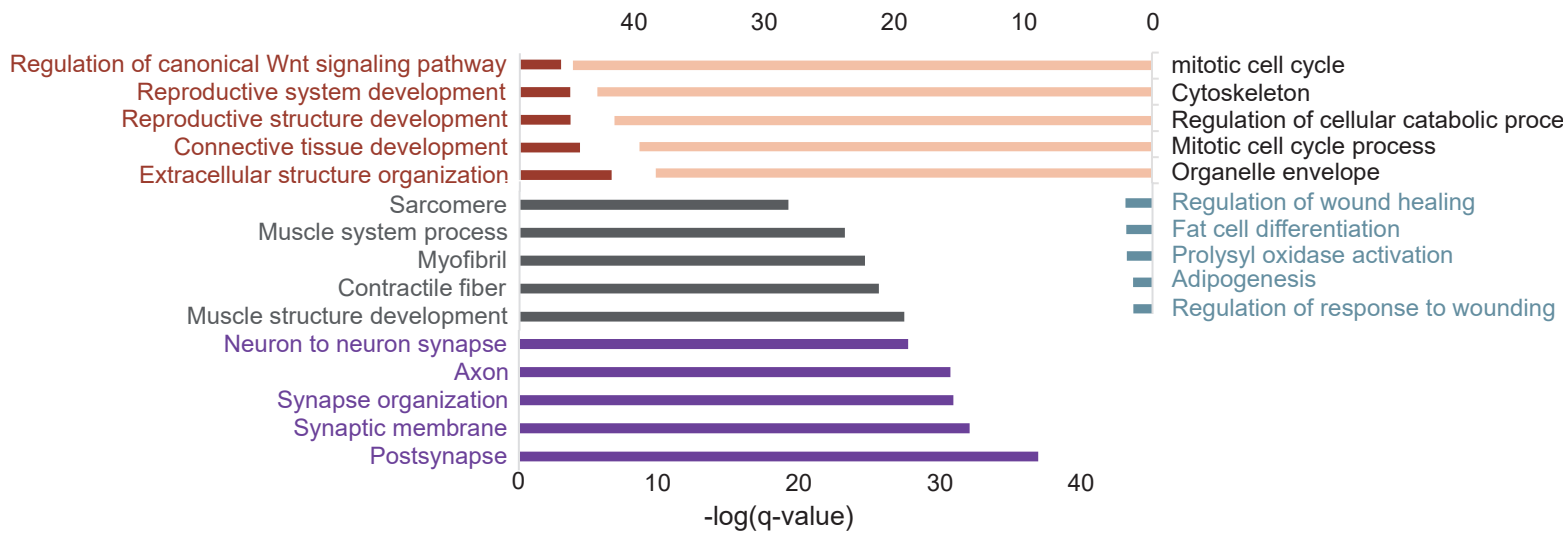**e**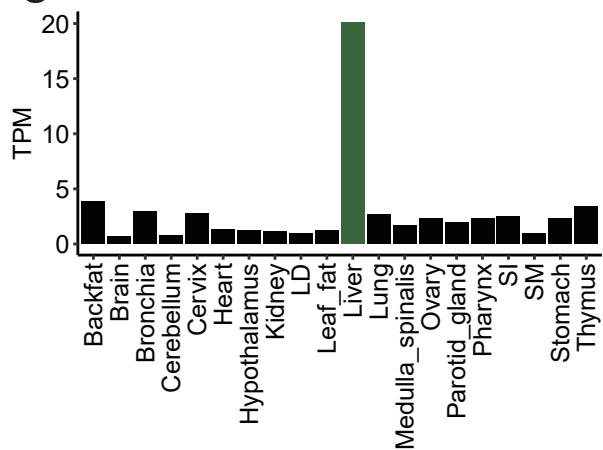**f**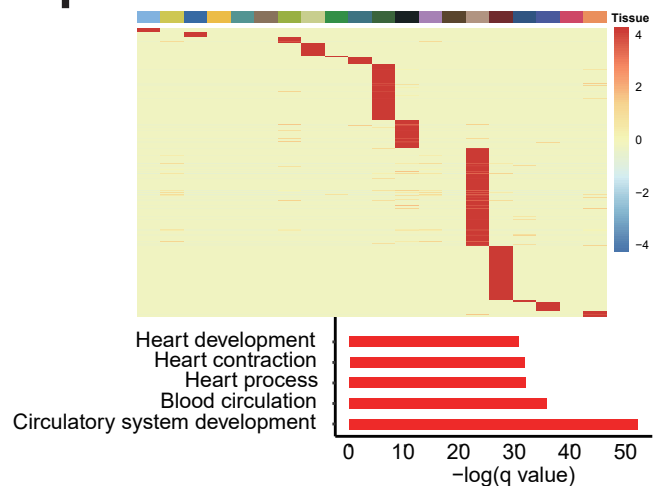

Supplement: Supplementary file 1 — Additional file 1: Table S1. Summary information on the ATAC-seq samples investigated in this study. Table S2. Summary information on the RNA-seq samples investigated in this study. Table S3. The information of download data. Table S4. Comparison with peaks of previous study designed by zhou et al. Table S5. Motif enrichment analysis of shared peaks. Table S6. The information of PERV overlapped peaks. Table S7. The information of GWAS SNPs which located in peak regions. Table S8. The information of virus data. Table S9. The information of virus data with sequence homology to shared open TEs. Table S10. GO and KEGG enrichment analysis of conserved accessible TEs homologous to viral sequences. [file 40104_2022_767_MOESM1_ESM.zip › Supplemental figure02.pdf]

ATAC-seq\_cluster

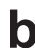

RNA-seq\_cluster

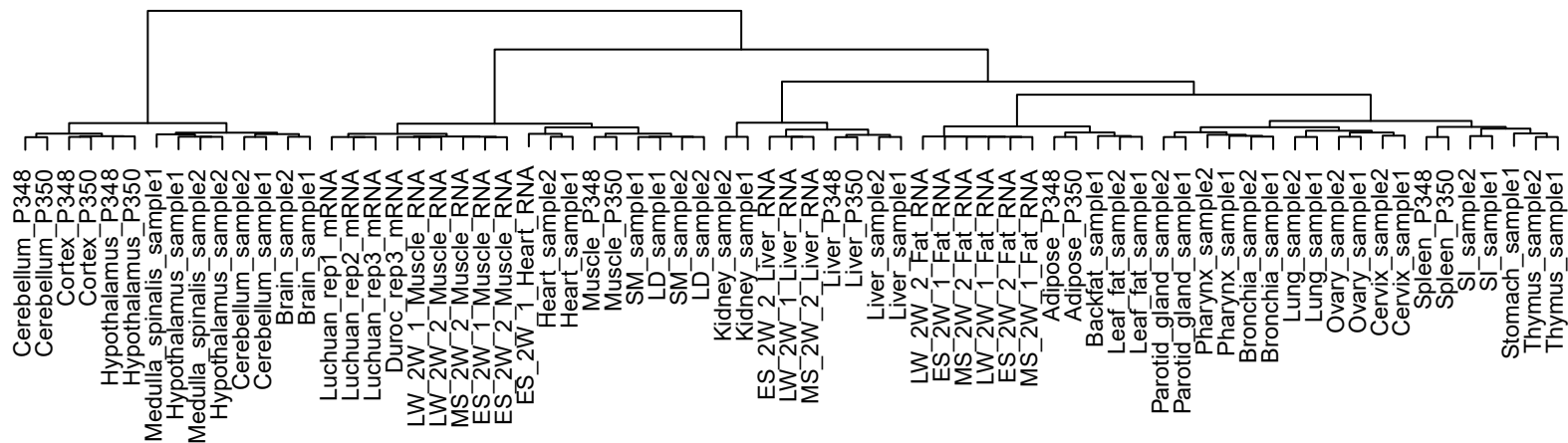

Supplement: Supplementary file 1 — Additional file 1: Table S1. Summary information on the ATAC-seq samples investigated in this study. Table S2. Summary information on the RNA-seq samples investigated in this study. Table S3. The information of download data. Table S4. Comparison with peaks of previous study designed by zhou et al. Table S5. Motif enrichment analysis of shared peaks. Table S6. The information of PERV overlapped peaks. Table S7. The information of GWAS SNPs which located in peak regions. Table S8. The information of virus data. Table S9. The information of virus data with sequence homology to shared open TEs. Table S10. GO and KEGG enrichment analysis of conserved accessible TEs homologous to viral sequences. [file 40104_2022_767_MOESM1_ESM.zip › Supplemental figure03.pdf]

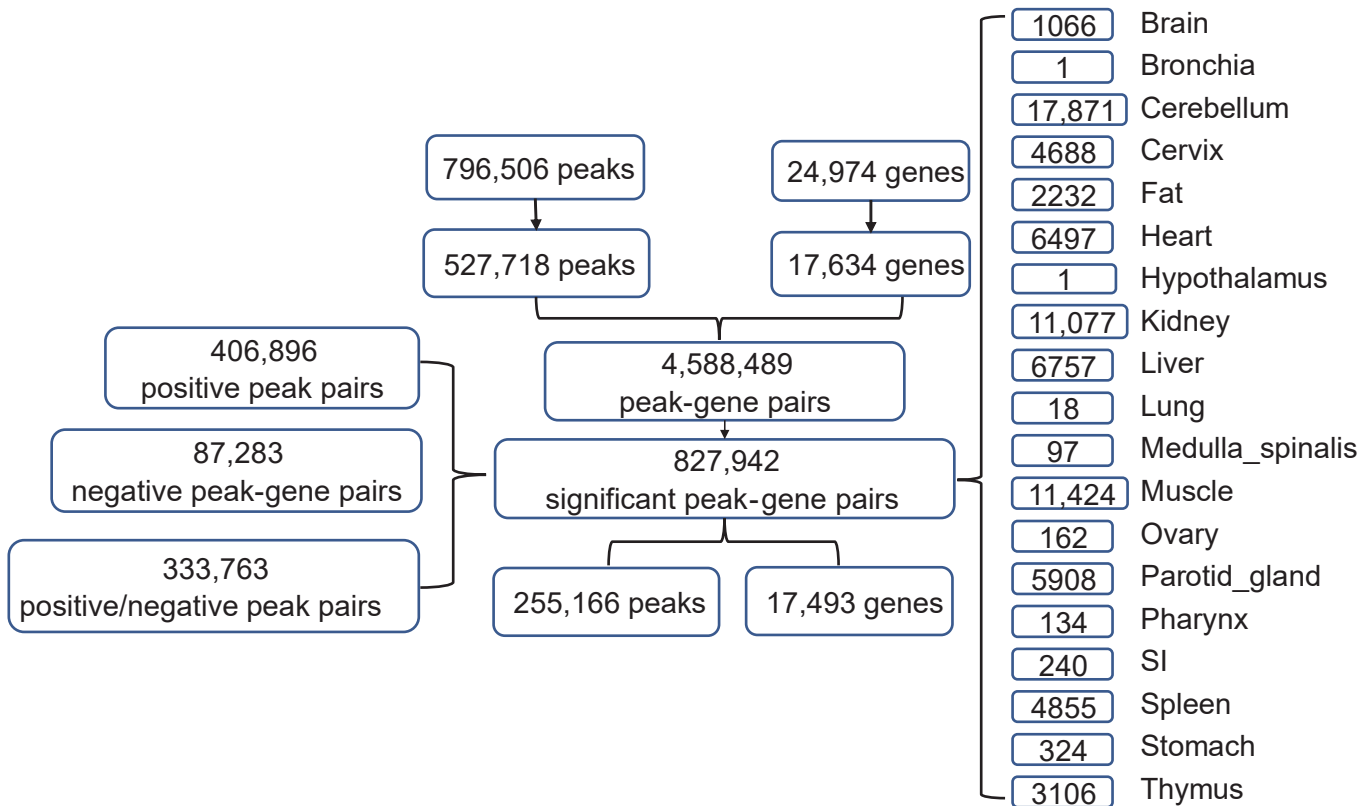

Supplement: Supplementary file 1 — Additional file 1: Table S1. Summary information on the ATAC-seq samples investigated in this study. Table S2. Summary information on the RNA-seq samples investigated in this study. Table S3. The information of download data. Table S4. Comparison with peaks of previous study designed by zhou et al. Table S5. Motif enrichment analysis of shared peaks. Table S6. The information of PERV overlapped peaks. Table S7. The information of GWAS SNPs which located in peak regions. Table S8. The information of virus data. Table S9. The information of virus data with sequence homology to shared open TEs. Table S10. GO and KEGG enrichment analysis of conserved accessible TEs homologous to viral sequences. [file 40104_2022_767_MOESM1_ESM.zip › Supplemental figure04.pdf]
